# Autoimmune Autonomic Dysfunction Syndromes: Potential Involvement and Pathophysiology Related to Complex Regional Pain Syndrome, Fibromyalgia, Chronic Fatigue Syndrome, Silicone Breast Implant–Related Symptoms and Post-COVID Syndrome

**DOI:** 10.3390/pathophysiology29030033

**Published:** 2022-07-28

**Authors:** Naim Mahroum, Yehuda Shoenfeld

**Affiliations:** 1 International School of Medicine, Istanbul Medipol University, Istanbul 34810, Turkey; 2 Ariel University, Ariel 4076414, Israel; yehuda.shoenfeld@sheba.health.gov.il

**Keywords:** autonomic nervous system, complex regional pain syndrome, fibromyalgia, chronic fatigue syndrome, silicone breast implants, post-COVID syndrome

## Abstract

The pathophysiological mechanisms involved in chronic disorders such as complex regional pain syndrome, fibromyalgia, chronic fatigue syndrome, silicone breast implant–related symptoms, and post-COVID syndrome have not been clearly defined. The course of the pain in some of the syndromes, the absence of evident tissue damage, and the predominance of alterations in the autonomic nervous system are shared similarities between them. The production of autoantibodies following a trigger in the syndromes was previously described, for instance, trauma in complex regional pain syndrome, infectious agents in fibromyalgia, chronic fatigue syndrome, and post-COVID syndrome, and the immune stimulation by silicone in women with breast implants. In fact, the autoantibodies produced were shown to be directed against the autonomic nervous system receptors, leading to the amplification of the perception of pain alongside various clinical symptoms seen during the clinical course of the syndromes. Therefore, we viewed autoantibodies targeting the autonomic nervous system resulting in autonomic dysfunction as likely the most comprehensive explanation of the pathophysiology of the disorders mentioned. Based on this, we aimed to introduce a new concept uniting complex regional pain syndrome, fibromyalgia, chronic fatigue syndrome, silicone breast implant–related symptoms, and post-COVID syndrome, namely “autoimmune autonomic dysfunction syndromes”. Due to its etiological, pathophysiological, and clinical implications, the suggested term would be more precise in classifying the syndromes under one title. The new title would doubtlessly facilitate both laboratory and clinical studies aimed to improve diagnosis and make treatment options more directed and precise.

## 1. Introduction

The interplay between pain processing and the autonomic nervous system has long been described [1]. Defined as unpleasant sensation or emotional experience associated with tissue damage by the international association for the study of pain (IASP) [2], the definition of pain has received various criticisms over the last decades. Among others, the need of regular updating due to the advances in researching pain at both physical and psychological levels [3,4], and the addition of cognitive and social components of pain to the definition [5] have been suggested. While the definition is still controversial [6], the debate and research in the field of pain etiology are far more complicated, especially when it comes to chronic pain without apparent tissue damage or inflammation. For instance, the pathophysiology of chronic pain syndromes such as complex regional pain syndrome, chronic fatigue syndrome, and fibromyalgia remains unestablished. In this regard, Goebel addressed the mechanism of pain as “autoantibody pain”, secondary to the Fc-region binding of autoantibodies found in chronic pain syndromes and thus modifying the release of neurotransmitters resulting in various intensities of pain [7]. The theory of autoantibody pain was later strengthened by Cuhadar et al. [8] in an animal-based experiment. The study included skin incision in mice followed by the administration of pathological autoantibodies (IgG) from patients with complex regional pain syndrome. Mice treated with the IgG showed hypersensitivity to both cold and heat stimulation compared to mice treated with IgG from healthy individuals. Mice with skin incision alone without IgG administration manifested the same findings. Moreover, the symptomatology in women with silicone breast implants has shown various autoimmune features in terms of autoantibody production and clinical presentation. The basis for silicone breast implant illness (BII) was formerly reviewed [9]. A causal association between the systemic reaction to the implants as in autoimmune/autoinflammatory syndrome induced by adjuvants (ASIA), the production of autoantibodies, the resultant symptoms including autonomic dysautonomia and cognitive impairment, and eventually the disappearance of symptoms following the removal of the implants, have all been addressed. Recently, the proposed and yet undefined mechanisms behind the post-COVID or long COVID syndrome, characterized by nonspecific general complaints following clinical recovery from COVID-19, brought to our attention the similarities between post COVID and other syndromes such as chronic fatigue syndrome and fibromyalgia [10]. The suggestion that a combination of biological, psychological, and social factors are involved in post-COVID pathophysiology [11] strengthened our assumption of a common mechanism between the entities. In addition, the presence of functional autoantibodies against G-protein coupled receptors (GPCR) related to the autonomic nervous system in patients with persistent post-COVID symptoms, particularly neurological complaints [12], also supports our hypothesis that autoimmunity is a broad pathophysiological base of the syndromes.

**Figure 1:**
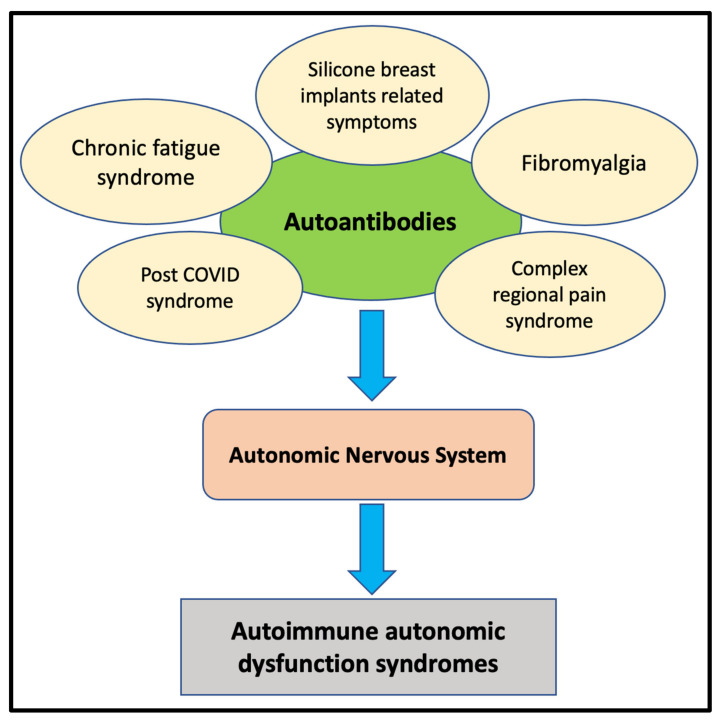
The presence of autoantibodies directed against the autonomic nervous system receptors and the subsequent clinical presentations of the syndromes.

Correspondingly, based on previous studies addressing autoimmune aspects of the syndromes, we introduce in this review a new concept in regard to the pathophysiological mechanism of the aforementioned chronic syndromes, called “autoimmune autonomic dysfunction syndromes” (Figure 1). In our opinion, the concept is wider, taking into consideration the clinical aspects shared by complex regional pain syndrome, chronic fatigue syndrome, fibromyalgia, silicone breast implant–related symptoms, and post-COVID syndrome. Our proposal is grounded and strengthened in the current paper.

## 2. Complex Regional Pain Syndrome (CRPS)

CRPS is a chronic pain disorder of undefined etiology characterized by persistent pain confined to one region of the body, more commonly affecting the lower extremities [13]. In addition to pain, motor, sensory, and autonomic abnormalities are observed [14]. The majority of patients with CRPS have no peripheral nerve injury and are categorized as type I CRPS, whereas a small percentage of patients suffering peripheral nerve injury are referred to as type II CRPS [15].

Due to the unanswered questions regarding the etiology and pathophysiology of the disease, CRPS has been widely investigated in the last decades [16]. For instance, in a systematic review and meta-analysis, Parkitny and colleagues [17] showed a significant increase in serum inflammatory cytokines such as interleukin (IL)-8 and soluble tumor necrosis factor (TNF) in the acute phase of the disease. In turn, in chronic disease, TNF-alpha and bradykinins, among others, were elevated in blisters and cerebrospinal fluid (CSF). In addition, a decrease in substance P, E- and L-selectin was observed. The authors concluded that patients with CRPS show a proinflammatory state manifested by high inflammatory cytokines in the blood, blisters, and CSF, with several differences in the acute and chronic course of the disease. Similar findings supporting a proinflammatory condition were documented by Alexander et al. [18], who demonstrated significantly high CSF levels of IL-1beta, IL-6, and TNF-alpha in patients with CRPS compared to individuals with other pain syndromes as well as healthy subjects.

Furthermore, the role of the autonomic nervous system in the manifestation of CRPS has been widely documented yet not fully established. Birklein and colleagues [19] thoroughly investigated autonomic dysfunction in patients with CRPS in both the acute and chronic stages of the disease. Patients in the study were examined twice, shortly after diagnosis and 2 years afterward. Skin temperature was found warmer in the acute stage and colder in the chronic stage, whereas sweating was increased in both stages; all were statistically significant. More recently, alteration in the autonomic nervous system, particularly the sympathetic one, and its implication in the pathophysiology of CRPS were demonstrated [20]. In fact, the autonomic nervous system was viewed as a target of autoantibodies resulting in CRPS. Autoantibodies against receptors in the autonomic nervous system were found in 40% of patients with CRPS, suggesting an autoimmune nature of the disease [21]. Moreover, functional autoantibodies of IgG directed against beta-2 and muscarinic-2 receptors were detected in the serum of patients with CRPS [22]. The autoantibodies isolated showed pain-like properties. In another study comparing sera from patients with CRPS, rheumatoid arthritis, axial spondyloarthritis, psoriatic arthritis, and healthy individuals, the specificity and sensitivity of P29ING4 autoantibodies were found to be higher in patients with CRPS [23]. As a result, the authors suggested to use the immune complexes of P29ING4 in the diagnostic work-up in patients suspected to suffer from CRPS. Therefore, and unsurprisingly in our view, CRPS was suggested to be an autoimmune disease by Goebel et al. [24]. Due to the correlation between trauma and the appearance of CRPS, the authors proposed a term called “IRAM”, indicating an injury-triggered, regionally restricted, autoantibody-mediated autoimmune disorder with minimally destructive course. Moreover, when skin incisions in mice were treated with IgG isolated from patients with longstanding CRPS, the skin showed prolonged swelling and hyperalgesia in comparison to the control group. In addition, mice treated with IgG from patients with CRPS manifested cellular changes such as microglia and astrocyte activation at the dorsal horn as well as pain regions in the brain [25]. The findings were supported by a recent study by Sahbaie and colleagues [26]. Passive transfer of serum from mice with induced fracture containing autoantibodies against the autonomic nervous system receptors and the role in pain processing were interestingly presented. The authors concluded that targeting the autonomic nervous system might aid in the prevention and treatment of chronic pain following trauma such as CRPS.

## 3. Fibromyalgia

A generalized and chronic pain is a central component in the definition of fibromyalgia, in addition to fatigue as well as cognitive and sleep disorders [27]. The etiology of fibromyalgia is still unclear; however, the correlation between infectious agents and the development of fibromyalgia is well documented in the medical literature [28,29,30]. In recent years, the central nervous system (CNS) was shown to serve as a key factor in the pathogenesis of fibromyalgia. Abnormalities in pain processing and neurochemical imbalances in the CNS were found to result in central amplification of pain in patients with fibromyalgia [31]. Furthermore, studies on structural and functional brain magnetic resonance imaging (MRI) in patients with fibromyalgia demonstrated several abnormalities in grey matter volume, the pain-modulating system, and the pain matrix [32]. The abnormalities of the CNS, with an emphasis on pain hypersensitivity, were postulated to occur secondary to autoimmune mechanisms. Buskila et al. [33] addressed the association and possible correlation between fibromyalgia and autoimmunity. Patients with fibromyalgia were found to have a variety of autoantibodies, including antinuclear antibody (ANA) as well as antithyroid antibodies. In addition, high incidence of fibromyalgia in patients with autoimmune and rheumatic diseases was illustrated. Furthermore, in an animal-based study, Goebel and colleagues [34] injected mice with IgG isolated from patients with fibromyalgia. Mice treated with IgG from fibromyalgia patients showed an amplified response to mechanical and cold stimulation compared to IgG from healthy people and IgG-depleted fibromyalgia patients. According to the study, the IgG isolated were found to hyperstimulate nociceptive afferents in the dorsal root ganglia responsible for the hypersensitivity to pain stimuli seen in fibromyalgia. Accordingly, we agree with Martinez-Lavin, who addressed fibromyalgia as possibly an autoimmune disease, stressing the importance of further investigations and the need for scientific-based evidence [35].

## 4. Chronic Fatigue Syndrome

Fatigue is a subjective feeling of tiredness leading to the inability to conduct regular activities [36]. In turn, “chronic fatigue syndrome” (CFS), or myalgic encephalomyelitis (ME), is a clinical condition based on several criteria aimed to better diagnose, investigate, and treat patients suffering from fatigue [37,38]. The history of fatigue and its related nomenclature, starting from “neuromyasthenia”, through “myalgic encephalomyelitis”, and finally “chronic fatigue syndrome”, was described in previous papers [39,40]. While fatigue is the predominant complaint of the syndrome, a chronic and generalized pain is common and often underestimated in patients with CFS [41]. Additionally, cognitive impairment, sleep disorders, as well as orthostatic-related symptoms were documented [39]. Regarding the etiology of CFS, the syndrome cannot be viewed as a disease caused by one factor; rather, CFS is in fact a multifactorial disorder induced by a combination of physiological and psychological causes [42]. Infections and infectious agents were correlated and addressed as a trigger for CFS [43]. Unsurprisingly, since the beginning of the COVID-19 pandemic [44], fatigue has been linked to COVID-19 in diverse manners. For example, fatigue was the most common manifestation of the post-COVID period in patients recovering from SARS-CoV-2 infection [45,46].

Therefore, the correlation and similarities between CFS, fibromyalgia, and post-COVID syndrome are numerous and may share a common mechanism. The involvement and disturbances of the autonomic nervous system in CFS has been described. Freeman and colleagues [47] reported significant alterations in the sympathetic and parasympathetic nervous system functions in 23 patients with CFS compared to healthy controls. During tilt tests conducted in the patients, alterations were recorded in expiratory to inspiratory ratio, systolic and diastolic blood pressure, and heart rate. Moreover, autonomic dysfunction was strongly associated with fatigue in 40 patients with CFS compared to healthy age- and sex-matched controls [48]. In addition, a recent study that enrolled 1226 patients with CFS suffering from cervical muscle-related complaints documented an involvement of the autonomic nervous system [49]. The recovery of patients after treatment with physical therapy was found to be linked to the amelioration of the autonomic nervous system function. The autonomic dysfunction described was found to be related to autoimmune mechanisms. Autoimmune etiology in CFS, including autoantibodies against neurotransmitters and their role in severe metabolic disturbances seen in patients with CFS, were previously reported [50].

## 5. Silicone Breast Implant (SBI)–Related Symptoms

The correlation between silicone breast implants and autoimmunity in terms of the production of autoantibodies and the clinical presentation is not a new finding. High titers of antinuclear antibodies (ANA) were found in 10 out of 11 symptomatic women with SBIs [51]. The characteristics of isolated ANA resembled autoantibodies found in idiopathic forms of autoimmune diseases. Furthermore, a thorough analysis of 20 autoantibodies in 116 women with SBIs compared to 134 controls was conducted by Bar-Meir et al. [52]. The main symptoms in the group of women with SBIs were fatigue, myalgia, polyarthralgia, and impaired memory. A significantly increased incidence of autoantibodies against 15 of the 20 autoantigens studied was detected in the breast implant group. Around 20% of the breast implant group had four autoantibodies, and 8% had six autoantibodies. In addition, Zandman-Goddard and colleagues [53] reported an increased incidence of autoantibody production in women with SBIs, particularly of anti-SSB/La and anticollagen II types, in both symptomatic and asymptomatic women. However, the authors showed that polyclonality of the autoantibodies was more prominent in the symptomatic group. A correlation between the duration of implants and the appearance of the autoantibodies was also demonstrated.

In regard to the function of the autonomic nervous system in women with SBIs, alterations in the autonomic nervous system and its dysfunction were termed “autoimmune dysautonomia” by Halpert et al. [54]. The authors suggested the name “autoimmune dysautonomia” following the detection of low sera levels of autoantibodies against G protein-coupled receptors (GCPRs) of the autonomic nervous system in 93 women with SBIs. Women enrolled in the study demonstrated a constellation of symptoms, including fatigue, dry mouth and eyes, and cognitive impairment. The results were compared to the levels of autoantibodies in 36 age-matched healthy women without silicone implants, enrolled as controls. A chronic stimulation of the immune system by the silicone implants resulting in autonomic dysautonomia and the subsequent symptoms was concluded by the authors. Since low levels of autoantibodies were detected in women with SBIs compared to controls, another assumption was that these autoantibodies might play an immunomodulatory role in the pathogenesis of the disease rather than a pathogenic role. However, the assumption requires further investigations to be confirmed; the authors are currently working on this hypothesis.

## 6. Post-COVID Syndrome

Post-COVID and long COVID have been used interchangeably to address a variety of symptoms in the period of time following the clinical recovery from acute SARS-CoV-2 infection [55]. Among others, the symptoms of post-COVID include respiratory, cardiovascular, digestive, and neurological complaints [56,57]. Brain tissues were found to be highly permeable to SARS-CoV-2 in contrast to SARS-CoV, the virus responsible for the SARS outbreak in 2002, shedding light on the neurological presentation of COVID-19 and proposing a possible explanation for the neurological manifestations of post-COVID syndrome [58]. Similarly, immune activation and neuroinflammation as a result of SARS-CoV-2 infection were also reported [59].

In fact, the role the immune system plays in the manifestations and complications of COVID-19 induced by hyperstimulation, hyperferritinemia with its pathological consequences, and the cytokine storm [60,61] cannot be overemphasized. As a result, the strong relation between COVID-19 and autoimmunity in terms of pathophysiology, presentation, complications, and treatment has been described previously [62,63]. Accordingly, the involvement of autoimmune factors in the pathogenesis of post-COVID syndrome was, to a large extent, predicted [64]. For instance, Wang et al. [65] reported a significantly high level of autoantibodies in patients with COVID-19 in comparison to healthy subjects. When injected in mice, the autoantibodies were shown to disturb mice immune function and exacerbate the course of the disease. Moreover, by investigating a total of 31 individuals with post-COVID complaints, mainly neurological ones, Walkut et al. [12] detected autoantibodies against G-protein coupled receptors (GPCR) in all the patients enrolled. The autoantibodies isolated were directed against the autonomic nervous system and showed both positive and negative chronotropic effects, depending on the targeted receptors: beta2-adrenorecptors or muscarinic receptors. Interestingly, nociception-like opioid receptors were targeted by the isolated autoantibodies.

Papers and studies addressing autoimmune mechanisms in the syndromes investigated are summarized in Table 1.

## 7. Conclusions

We view autoimmunity, in the form of autoimmune autonomic dysfunction as a potential pathophysiological mechanism of complex regional pain syndrome, fibromyalgia, chronic fatigue syndrome, silicone breast implant–related symptoms, and post-COVID syndrome, as revolutionary. As the pathophysiology of the syndromes addressed is unclear, the presence of autoantibodies directed against the autonomic nervous system receptors and the subsequent clinical presentations offer a comprehensive pathophysiological explanation. The term “autoimmune autonomic dysfunction syndromes” could serve as a base for directed research, initially through detailed animal model–based experiments, followed by appropriately designed clinical studies. The results will doubtlessly improve the diagnosis of the syndromes and provide targeted treatment options.

## Figures and Tables

**Table 1:** Papers and studies addressing autoimmune mechanisms in the syndromes investigated.

Syndrome	Authors	Name of study	Conclusion
**CRPS**	Birkelin et al. [19]	Pattern of autonomic dysfunction in time course of complex regional pain syndrome.	Alterations of ANS differ in acute and chronic stages of CRPS
	Knudsen et al. [20]	Complex regional pain syndrome: a focus on the autonomic nervous system.	Alterations in the sympathetic nervous system contribute to CRPS pathology.
	Blaes at al [21]	Autoimmune etiology of complex regional pain syndrome.	Autoantibodies against receptors in the autonomic nervous system were found in 40% of patients with CRPS, suggesting an autoimmune nature of the disease
	Kohr et al. [22]	Autoimmunity against the beta2 adrenergic receptor and muscarinic-2 receptor in complex regional pain syndrome.	functional autoantibodies of IgG directed against beta-2 and muscarinic-2 receptors were detected in the serum of patients with CRPS. The autoantibodies isolated showed pain-like properties
	Goebel et al. [24]	Complex regional pain syndrome, prototype of a novel kind of autoimmune disease.	The term IRAM” indicating injury-triggered, regionally restricted, autoantibody-mediated autoimmune disorder with minimally destructive course, was proposed.
	Helyes et al. [25]	Transfer of complex regional pain syndrome to mice via human autoantibodies is mediated by interleukin-1-induced mechanisms.	The skin of mice treated with IgG isolated from patients with longstanding CRPS showed prolonged swelling and hyperalgesia in comparison to the control group. In addition, mice treated with CRPS IgG manifested cellular changes as microglia and astrocyte activation at the dorsal horn as well as pain regions in the brain.
	Sahbaie et al. [26]	Autonomic Regulation of Nociceptive and Immunologic Changes in a Mouse Model of Complex Regional Pain Syndrome.	The ANS regulates adaptive immune system activation and nociceptive sensitization in a mouse model of chronic post-traumatic pain with features of CRPS.
**Fibromyalgia**	Clauw et al. [31]	The science of fibromyalgia	Abnormalities in pain processing and neurochemical imbalances in the CNS were found to result in central amplification of pain in patients with fibromyalgia.
	Cagnie et al. [32]	Central sensitization in fibromyalgia? A systematic review on structural and functional brain MRI	Structural and functional brain magnetic resonance imaging (MRI) in patients with fibromyalgia demonstrated several abnormalities in grey matter volume, pain-modulating system, and pain matrix.
	Buskila et al. [33]	Fibromyalgia and autoimmune diseases: the pain behind autoimmunity	An association and possible correlation between fibromyalgia and autoimmunity exist. Patients with fibromyalgia were found to have a variety of autoantibodies including antinuclear antibody (ANA) as well as antithyroid antibodies. In addition, high incidence of fibromyalgia in patients with autoimmune and rheumatic diseases was illustrated.
	Goebel et al. [34]	Passive transfer of fibromyalgia symptoms from patients to mice	Mice injected with IgG isolated from patients with fibromyalgia showed amplified response to mechanical and cold stimulation compared to IgG from healthy people and IgG-depleted fibromyalgia patients. According to the study, the IgG isolated was found to hyperstimulate nociceptive afferents in the dorsal root ganglia which are responsible for the hypersensitivity to pain stimuli seen in patients with fibromyalgia.
**CFS**	Freeman et al. [47]	Does the chronic fatigue syndrome involve the autonomic nervous system?	Significant alterations in the sympathetic and parasympathetic nervous system functions in 23 patients with CFS compared to healthy controls was registered. During tilt tests conducted in the patients, alterations were recorded in expiratory to inspiratory ratio, systolic and diastolic blood pressure, and heart rate.
	Newton et al. [48]	Symptoms of autonomic dysfunction in chronic fatigue syndrome	Autonomic dysfunction was strongly associated with fatigue in 40 patients with CFS compared to healthy age- and sex-matched controls.
	Matsui et al. [49]	Possible involvement of the autonomic nervous system in cervical muscles of patients with myalgic encephalomyelitis / chronic fatigue syndrome (ME/CFS)	The recovery of 1226 patients with CFS suffering from cervical muscle-related complaints was found to be linked to the amelioration of the ANS function.
	Sotzny et al. [50]	Myalgic Encephalomyelitis/Chronic Fatigue Syndrome - Evidence for an autoimmune disease	Autoimmune etiology of CFS, including autoantibodies against neurotransmitters and their role in severe metabolic disturbances seen in patients with CFS.
**SBIs**	Press et al. [51]	Antinuclear autoantibodies in women with silicone breast implants	High titers of antinuclear antibodies (ANA) were found in 10 out of 11 symptomatic women with silicone breast implants.
	Bar-Meir et al. [52]	Multiple autoantibodies in patients with silicone breast implants	A thorough analysis of 20 autoantibodies in 116 women with silicone breast implants compared to 134 controls. A significantly increased incidence of autoantibodies against 15 of the 20 autoantigens studied was detected in the breast implants group. Around 20% of the breast implants group had 4 autoantibodies, and 8% had 6 autoantibodies.
	Zandman-Goddard et al. [53]	A comparison of autoantibody production in asymptomatic and symptomatic women with silicone breast implants	An increased incidence of autoantibody production in women with silicone breast implants particularly of anti-SSB/La and anticollagen II types, both symptomatic and asymptomatic women. Polyclonality of the autoantibodies was more prominent in the symptomatic group.
	Halpert et al. [54]	Autoimmune dysautonomia in women with silicone breast implants	Chronic immune stimulation by silicone material may lead to an autoimmune dysautonomia in a subgroup of potentially genetically susceptible women with SBIs. The appearance of autoantibodies against GPCRs of the autonomic nervous system serve as an explanation for the subjective autonomic-related manifestations reported in women with SBIs.
**Post-COVID**	Zhang et al. [58]	SARS-CoV-2 infects human neural progenitor cells and brain organoids	Brain tissues were found to be highly permeable to SARS-CoV-2 shedding lights on the neurological presentation of COVID-19 and proposing a possible explanation for the neurological manifestations of the post-COVID syndrome.
	Kempuraj et al. [59]	COVID-19, Mast Cells, Cytokine Storm, Psychological Stress, and Neuroinflammation	COVID-19 can activate mast cells, neurons, glial cells, and endothelial cells. SARS-CoV-2 infection can cause psychological stress and neuroinflammation. In conclusion, COVID-19 can induce mast cell activation, psychological stress, cytokine storm, and neuroinflammation.
	Ehrenfeld et al. [62]	COVID-19 and autoimmunity	A strong relation between COVID-19 and autoimmunity in terms of pathophysiology, presentation, complications, and treatment.
	Lyons-Weiler J [63]	Pathogenic priming likely contributes to serious and critical illness and mortality in COVID-19 via autoimmunity	
	Damoiseaux et al. [64]	Autoantibodies and SARS-CoV2 infection: The spectrum from association to clinical implication	The relation between infections and autoimmune diseases has been suggest with molecular mimicry, hyperstimulation and dysregulation of the immune system as plausible mechanisms. The recent pandemic with a new virus, i.e., SARS-CoV-2, has resulted in numerous studies addressing the potential of this virus to induce autoimmunity and, eventually, autoimmune disease. In addition, it has also revealed that pre-existing auto-immunity (auto-Abs neutralizing type I IFNs) could cause life-threatening disease.
	Wang et al. [65]	Diverse functional autoantibodies in patients with COVID-19	Our analysis of autoantibodies against tissue-associated antigens revealed associations with specific clinical characteristics. Our findings suggest a pathological role for exoproteome-directed autoantibodies in COVID-19, with diverse effects on immune functionality and associations with clinical outcomes.

## Data Availability

Not applicable.
